# Associations Between Serum 25(OH)D Concentrations and Clinical Characteristics in Pediatric Patients

**DOI:** 10.3390/reports9010054

**Published:** 2026-02-09

**Authors:** Maria Nicolae, Sorin Deacu, Cristina Maria Mihai, Tatiana Chisnoiu, Irina Ion, Claudia Simona Cambrea, Andreea Nelson Twakor, Alexandra Herlo, Oana Cristina Arghir

**Affiliations:** 1 Doctoral School, Faculty of General Medicine, “Ovidius” University, 900470 Constanta, Romania; dr.nicolae_maria@yahoo.com (M.N.); iryna_ion@yahoo.com (I.I.); 2 Department of Forensic Medicine, County Clinical Emergency Hospital of Constanta, 900591 Constanta, Romania; 3 Department of Paediatrics, Faculty of General Medicine, “Ovidius” University, 900470 Constanta, Romania; cristina_mihai@365.univ-ovidius.ro (C.M.M.); tatiana_ceafcu@yahoo.com (T.C.); 4 Department of Infectious Diseases, Faculty of General Medicine, “Ovidius” University, 900470 Constanta, Romania; cambrea.claudia@gmail.com; 5 Internal Medicine Department, “Sf. Apostol Andrei” Emergency County Hospital, 145 Tomis Blvd., 900591 Constanta, Romania; andreea.purcaru@365.univ-ovidius.ro; 6 Department XIII, Discipline of Infectious Diseases, “Victor Babes” University of Medicine and Pharmacy Timisoara, 2 Eftimie Murgu Square, 300041 Timisoara, Romania; alexandra.mocanu@umft.ro; 7 Clinical Pneumology Hospital, 40 Santinelei Str., 900002 Constanta, Romania; arghir_oana@yahoo.com

**Keywords:** vitamin D, hypovitaminosis D, pediatric hospitalization, length of stay, serum 25(OH)D, nutritional deficiency, pediatric patients, hospital outcomes, immune function, inpatient care

## Abstract

**Background/Objectives:** Vitamin D has an essential role in immune modulation and inflammatory control, particularly in respiratory infections. Despite widespread supplementation policies, hypovitaminosis D remains common in children and data linking vitamin D status to hospitalization outcomes in pediatric upper respiratory tract infections are limited, especially in Eastern Europe. **Methods:** We included 400 pediatric patients hospitalized between October 2020 and December 2024 for acute respiratory tract infections (ARTI), and we stratified them into a Normal Vitamin D group (NVD) with sufficient serum 25(OH)D concentrations and a Low Vitamin D group (LVD) with insufficient or deficient levels. Between-group comparisons for continuous variables were performed using non-parametric methods. **Results:** Children with insufficient or deficient 25(OH)D had a significantly longer duration of hospitalization compared with those with sufficient levels (mean 4.68 ± 2.59 days vs. 2.89 ± 1.81 days). The LVD group showed markedly lower serum vitamin D concentrations (mean 21.63 ± 5.56 ng/mL; median 22.29 ng/mL) compared with the NVD group (mean 47.60 ± 19.59 ng/mL; median 43.70 ng/mL). Markers of disease severity were consistently higher in vitamin D-deficient patients, including higher clinical scores (mean 3.77 ± 2.29 vs. 1.62 ± 1.89), elevated CRP levels (mean 3.50 ± 3.02 mg/L vs. 1.64 ± 1.59 mg/L), and increased O_2_ therapy requirement (69.5% vs. 21.0%). Fever was more frequent in the LVD group (61.0% vs. 32.0%). An inverse correlation was observed between serum 25(OH)D concentrations and hospitalization duration, clinical score, and disease severity, with deficiency present across all age strata in the LVD group, while no cases of deficiency were observed in the NVD group. **Conclusions:** Low serum 25(OH)D concentrations are associated with increased disease severity and prolonged hospitalization.

## 1. Introduction

Vitamin D is an essential fat-soluble micronutrient with critical physiological roles extending beyond its classical involvement in calcium-phosphorus homeostasis and bone mineralization [1]. Over the past two decades, an expanding body of literature has emphasized its multifaceted contribution to immune regulation, cellular differentiation, neuromuscular function, and modulation of inflammatory responses [2]. In pediatric populations, adequate 25(OH)D is indispensable not only for the prevention of rickets and osteomalacia but also for optimal growth, immune competence, and neurodevelopmental health [3].

**Figure 1:**
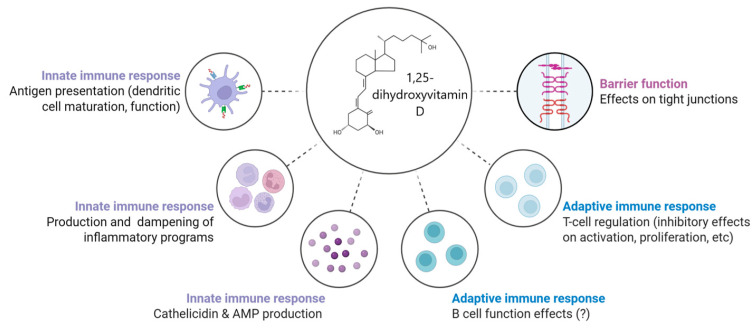
Multifaceted immunomodulatory roles of 1,25-dihydroxyvitamin D. Created with Biorender version 2.0 [7].

Emerging research has illuminated a strong association between vitamin D deficiency and increased susceptibility to infections, particularly of the respiratory tract, in children [4]. Additionally, vitamin D insufficiency has been linked to higher risks of autoimmune conditions, poor recovery from illness, and even prolonged hospitalization in some adult and neonatal cohorts [5]. While global awareness regarding the importance of maintaining adequate serum 25(OH)D concentrations has increased, the prevalence of hypovitaminosis D remains alarmingly high, especially among vulnerable pediatric groups [6]. Figure 1 shows the immunomodulatory roles of 1,25-dihydroxyvitamin D.

This diagram highlights the diverse and integral effects of vitamin D on various components of the immune system. Vitamin D influences both the innate and adaptive immune responses, as well as maintaining epithelial barrier function. In innate immunity, it enhances antigen presentation by regulating dendritic cell maturation, stimulates the production of antimicrobial peptides like cathelicidin, and modulates inflammatory pathways by either amplifying or suppressing inflammatory responses [8]. In the adaptive immune system, vitamin D plays a regulatory role by inhibiting T-cell activation and proliferation, and may affect B-cell differentiation and antibody production, although the latter remains less clearly defined [9]. Additionally, vitamin D contributes to barrier integrity by supporting tight junction function, which is crucial for preventing pathogen translocation across mucosal surfaces [10]. These effects demonstrate vitamin D’s immune gate-keeping significance, especially in paediatric health and hospital recovery.

In Eastern Europe, and particularly in Romania, hypovitaminosis D is a prevalent and often underdiagnosed public health concern. Several factors contribute to this phenomenon, including limited sun exposure during long winters, high rates of exclusive breastfeeding without vitamin D supplementation, urbanization, air pollution, dietary insufficiencies, and socio-economic disparities [11]. Despite nationwide prophylaxis recommendations, studies have reported suboptimal adherence and inadequate dosing practices, especially in underserved communities [12]. Comorbidities, severe infections, and poor baseline nutritional status may exacerbate vitamin D insufficiency in hospitalised children.

Although the link between vitamin D status and pediatric morbidity has been explored in various international settings, regional data from Romania and surrounding areas remain sparse [13]. Particularly lacking is evidence on how serum 25(OH)D concentrations correlate with key clinical outcomes such as the length of hospital stay—a parameter often reflective of disease severity, recovery trajectory, and resource utilization [13].

This study aims to fill this knowledge gap by analyzing a cohort of pediatric inpatients admitted to the Municipal Medgidia Hospital and Emergency County Hospital in Constanța. Using a retrospective dataset comprising demographic, clinical, and biochemical variables, the primary objective is to evaluate the association between serum 25(OH)D concentrations and the duration of hospitalization.

To the best of our knowledge, this study represents the first pediatric cohort from Eastern Romania to investigate the association between serum 25(OH)D concentrations and hospitalization duration across a wide spectrum of infectious and non-infectious diseases. While previous studies have explored this relationship in specific diagnostic subgroups, most often respiratory or COVID-19 infections, our work expands the analysis, thereby providing a broader clinical perspective on the potential role of vitamin D status in pediatric recovery.

Accordingly, the objective of this study was to evaluate whether lower serum 25(OH)D concentrations are associated with longer hospital stays in children admitted for respiratory tract infections. We hypothesized that pediatric patients with hypovitaminosis D (vitamin D < 30 ng/mL) would experience significantly longer hospitalization durations compared to those with sufficient levels, regardless of the underlying etiology.

## 2. Materials and Methods

### 2.1. Study Design and Setting

This retrospective observational study was conducted using pediatric hospitalization data collected at the Municipal Medgidia Hospital and Emergency County Hospital in Constanța, Romania. The study aimed to investigate the relationship between vitamin D and the duration of hospital stay among pediatric patients. The analysis was performed on anonymized data extracted from hospital records spanning from October 2020 to December 2024. The study protocol was reviewed and approved by the Ethics Committee of the Municipal Medgidia Hospital (approval no. 152/06.01.2022) and of the “Sf. Apostol Andrei” County Emergency Hospital, Constanța (approval no. 2770/16.01.2024). The research complied with the Declaration of Helsinki, and informed consent was waived due to the retrospective design and anonymization of patient data.

### 2.2. Study Population

Inclusion criteria were:

- Pediatric patients (aged 0–18 years) hospitalized for ARTIs.
- Availability of complete laboratory data, including serum vitamin D measurement.
- Clearly documented duration of hospitalization (in days).

Exclusion criteria were:

- Missing or incomplete data on vitamin D level, hospitalization duration, or demographic information.
- Known chronic inflammatory diseases, autoimmune disorders, malignancies, or immunodeficiency syndromes that could independently influence vitamin D metabolism or length of hospitalization.
- Endocrine or metabolic disorders (e.g., thyroid dysfunction, rickets under active treatment) that might alter vitamin D status.
- Re-hospitalizations of the same patient within the study period (to avoid duplication).
- Hospitalizations shorter than 24 h (to exclude brief observation cases).

During the study period, a total of 1732 pediatric patients were admitted to the pediatric department, of which 400 met the inclusion criteria for vitamin D testing and were included in the final analysis. The age of the children ranged from infancy to adolescence, and both sexes were represented. Data was stratified to analyze the prevalence and impact of hypovitaminosis D across different subgroups. Serum 25(OH)D concentrations were measured in patients for whom clinical suspicion of deficiency or related metabolic imbalance was present, as part of the hospital’s diagnostic evaluation protocol upon admission. Testing was thus performed selectively rather than systematically, depending on the treating physician’s judgment and the clinical presentation. Because the dataset was anonymized, information regarding the exact season of sampling and detailed clinical indications for vitamin D testing could not be retrieved. These factors are recognized as limitations of the retrospective design.

### 2.3. Variables and Definitions

We defined the NVD group as the group with sufficient serum 25(OH)D concentrations, and the LVD group as the group with insufficient and deficient concentrations.

Primary Outcome Variable:

Hospitalization duration (number of days): defined as the total number of inpatient days, calculated from admission to discharge dates.

Primary Independent Variable:

Vitamin D level (ng/mL): obtained from laboratory records.

Vitamin D status was classified into three categories based on Endocrine Society guidelines published by Holick et al. [14]:

Deficiency: <20 ng/mL.

Insufficiency: 20–30 ng/mL.

Sufficiency: >30 ng/mL.

Covariates and Potential Confounders:

Age (in years).

Sex (male/female).

Environment (urban/rural).

Ethnicity (Romanian, Turkish, Tatar, Macedonian, Ukrainian).

Presence in collectivity (e.g., daycare/school exposure).

Feeding type (natural, formula, mixed).

Vitamin D supplementation status (yes/no).

Additional laboratory markers where available: hemoglobin (Hb), serum iron, PTH, TSH, FT4, total IgE, phosphorus.

### 2.4. Data Collection and Processing

All data were collected from the electronic medical records and formatted into an SPSS-compatible spreadsheet. Variables were coded appropriately (e.g., binary coding for sex, environment, supplementation). Outliers and missing values were assessed; cases with critical missing data for primary variables were excluded. For some biochemical markers available only in subsets of patients, analyses were performed separately.

Descriptive statistics were used to summarize the study population. Continuous variables were assessed for distribution using descriptive measures and visual inspection, including histograms and boxplots. As most variables showed non-normal distributions, results are reported as medians with interquartile ranges or means with standard deviations where appropriate. Categorical variables were expressed as absolute frequencies and percentages.

Patients were stratified into two groups based on serum vitamin D status (>30 ng/mL vs. ≤30 ng/mL). Between-group comparisons for continuous variables, including hospitalization duration, serum 25(OH)D concentrations, inflammatory markers, and clinical scores, were performed using non-parametric methods. The Mann–Whitney U test was applied for two-group comparisons, while the Kruskal–Wallis test was used when comparisons involved more than two severity categories [15]. Categorical variables were compared using the chi-square test [16].

Associations between serum 25(OH)D concentrations and clinical parameters, including hospitalization duration, clinical score, CRP, age, and disease severity, were evaluated using Spearman’s rank correlation coefficient. Given the non-normal data distribution, Pearson correlation analysis was not applied.

A multivariable linear regression model was additionally explored to assess the independent relationship between serum 25(OH)D concentrations and hospitalization duration after adjustment for potential confounders, including age, vitamin D supplementation status, and feeding type. Statistical significance was defined as a two-sided *p* value < 0.05 [17]. All analyses were performed using IBM SPSS Statistics version 26.0 (IBM Corp., Armonk, NY, USA).

## 3. Results

The study population consisted of 400 pediatric patients, evenly distributed between the NVD group (*n* = 200) and the LVD group (*n* = 200). All analyzed variables had complete data, with no missing values in either group. The NVD group was characterized by a younger population, with a mean age of 4.43 ± 4.37 years and a median age of 2.0 years, compared with a higher mean age of 5.79 ± 4.73 years and a median of 5.0 years in the LVD group. Hospitalization duration differed markedly between groups, with the NVD group exhibiting a shorter mean length of stay of 2.89 ± 1.81 days (median 2 days), whereas patients in the LVD group required significantly longer hospitalization, with a mean duration of 4.68 ± 2.59 days and a median of 4 days. More population characteristics are included in Supplementary Materials. Table 1 shows the characteristics of NVD group.

**Table 1:** Population characteristics of the NVD group.

NVD Group								
		Age	Days of Hospitalisation	25(OH)D	CRP	Clinical Score	Total IgE	Hb
N	Valid	200	200	200	200	200	200	200
	Missing	0	0	0	0	0	0	0
Mean		4.43	2.89	47.5953	1.643	1.62	52.852	12.418
Median		2.00	2.00	43.7000	1.000	1.00	10.550	12.550
Std. Deviation		4.373	1.813	19.59063	1.5919	1.888	214.1844	1.2581
Range		14	10	179.90	8.4	7	2571.6	5.8
Minimum		1	1	30.10	0.1	0	0.4	9.2
Maximum		15	11	210.00	8.5	7	2572.0	15.0

Serum 25(OH)D concentrations showed a pronounced contrast between the two groups. The NVD group demonstrated substantially higher 25(OH)D concentrations, with a mean value of 47.6 ± 19.6 ng/mL and a median of 43.7 ng/mL, compared to markedly lower levels in the LVD group, where the mean was 21.6 ± 5.6 ng/mL, and the median was 22.3 ng/mL. Inflammatory status also differed between groups, as reflected by C-reactive protein levels. The NVD group had lower CRP values, with a mean of 1.64 ± 1.59 mg/L and a median of 1.0 mg/L, whereas the LVD group exhibited higher inflammatory markers, with a mean CRP of 3.50 ± 3.02 mg/L and a median of 2.85 mg/L. Table 2 shows the population characteritstics of LVD group.

**Table 2:** Population characteristics of the LVD group.

LVD Group								
		Age	Days of Hospitalisation	25(OH)D	CRP	Clinical Score	Total IgE	Hb
N	Valid	200	200	200	200	200	200	200
	Missing	0	0	0	0	0	0	0
Mean		5.79	4.68	21.6272	3.504	3.77	99.410	11.447
Median		5.00	4.00	22.2900	2.850	4.00	68.450	11.300
Std. Deviation		4.734	2.593	5.55887	3.0244	2.288	140.9638	1.7791
Range		15	15	26.04	23.8	7	1083.8	12.2
Minimum		1	1	3.96	2	0	1.2	4.4
Maximum		16	16	30	24	7	1085.0	16.6

Clinical severity indicators further distinguished the two populations. The mean clinical score in the NVD group was 1.62 ± 1.89, with a median score of 1.0, while the LVD group presented with significantly higher severity, reflected by a mean score of 3.77 ± 2.29 and a median of 4.0. Total IgE levels were also higher in the LVD group, with a mean value of 99.4 ± 141.0 IU/mL and a median of 68.5 IU/mL, compared to the NVD group, which showed a mean IgE of 52.9 ± 214.2 IU/mL and a median of 10.6 IU/mL, indicating greater atopic or inflammatory burden in the LVD cohort. Hemoglobin levels were modestly lower in the LVD group (mean 11.45 ± 1.78 g/dL; median 11.3 g/dL) compared to the NVD group (mean 12.42 ± 1.26 g/dL; median 12.55 g/dL). Table 3 shows the population frequencies of NVD group.

**Table 3:** Population frequencies of the NVD group.

NVD Group							
		Female	Rural	Romanians	Yes to Vitamin D Supplementation	Yes to Fever	Yes to O_2_ Therapy
N	Valid	200	200	200	200	200	200
	Missing	0	0	0	0	0	0
Total		97	67	142	102	64	42
% of total		48.5	33.5	71	51	32	21
Skewness		0.060	0.704	1.198	−0.040	0.778	1.435
Std. Error of Skewness		0.172	0.172	0.172	0.172	0.172	0.172
Kurtosis		−2.017	−1.519	−0.324	−2.019	−1.410	0.059
Std. Error of Kurtosis		0.342	0.342	0.342	0.342	0.342	0.342

Sex distribution showed a slight predominance of male patients in both cohorts. In the NVD group, 97 patients (48.5%) were female, compared to 82 patients (41.0%) in the LVD group. Rural residence was similarly distributed between groups, being reported in 67 NVD patients (33.5%) and 65 low Vitamin D patients (32.5%). The majority of patients in both groups were Romanian, accounting for 149 patients (74.5%) in the NVD group and 129 patients (64.5%) in the second study group. Prior vitamin D supplementation was more frequently reported among NVD patients, with 102 children (51.0%) receiving supplementation, compared to only 67 patients (33.5%) in the LVD group. Table 4 shows the population frequencies of LVD group.

**Table 4:** Population frequencies of the LVD group.

LVD Group							
		Female	Rural	Romanians	Yes to Vitamin D Supplementation	Yes to Fever	Yes to O_2_ Therapy
N	Valid	200	200	200	200	200	200
	Missing	0	0	0	0	0	0
Total		82	65	141	67	139	122
% of total		41	32.5	70.5	33.5	69.5	61
Skewness		0.369	0.753	1.436	0.704	−0.454	−0.853
Std. Error of Skewness		0.172	0.172	0.172	0.172	0.172	0.172
Kurtosis		−1.883	−1.448	0.564	−1.519	−1.812	−1.285
Std. Error of Kurtosis		0.342	0.342	0.342	0.342	0.342	0.342

Marked differences were observed in acute clinical manifestations and supportive therapy requirements. Fever was documented in 64 patients (32.0%) in the NVD group, whereas it was present in 122 patients (61.0%) in the LVD group, reflecting a considerably higher burden of acute illness among the latter. Similarly, the need for oxygen therapy was significantly more frequent in the LVD group, where 139 patients (69.5%) required supplemental oxygen, compared to only 42 patients (21.0%) in the NVD group.

### 3.1. Vitamin D Status and Systemic Inflammation

**Figure 2:**
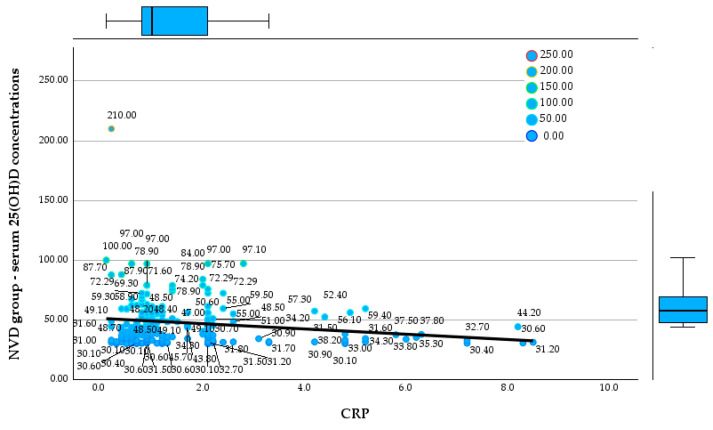
Relationship between serum 25(OH)D concentrations and CRP in the NVD group.

**Figure 3:**
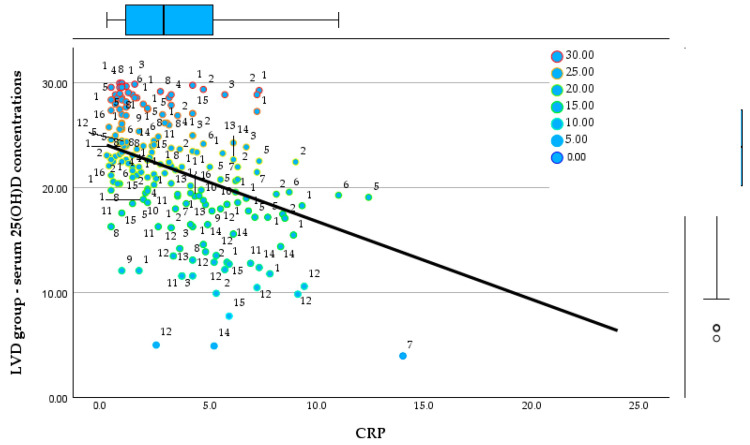
Relationship between serum 25(OH)D concentrations and CRP in the LVD group.

In the NVD group, serum 25(OH)D concentrations had a mean of 47.6 ng/mL and a median of 43.7 ng/mL, but with very wide dispersion (30.1–210 ng/mL). This high variability suggests the presence of distinct clinical subgroups, including children with adequate 25(OH)D levels as well as cases with extremely elevated values. In the LVD group, the fact that the descriptive analysis subsequently focuses on skewness and kurtosis suggests that the distribution of 25(OH)D levels is influenced by the intervention itself rather than solely by natural biological variability. Figure 2 and Figure 3 display the relationships between 25(OH)D concentrations and CRP in the NVD and LVD groups.

**Figure 4:**
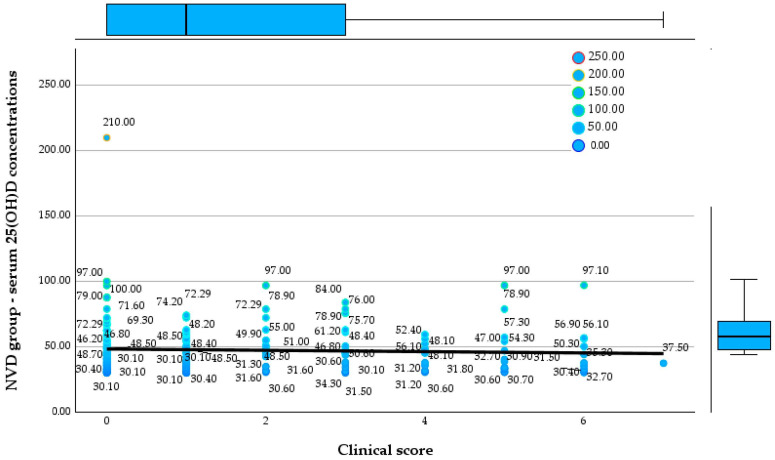
Relationship between serum 25(OH)D concentrations and clinical score in the NVD group.

**Figure 5:**
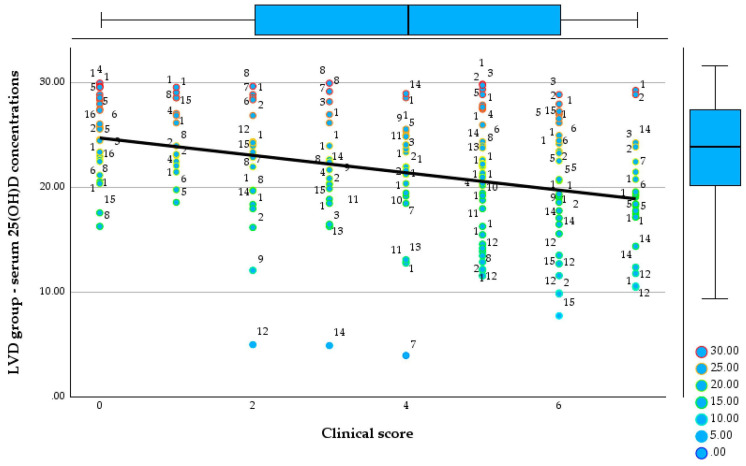
Relationship between serum 25(OH)D concentrations and clinical score in the LVD group.

Regarding CRP, the NVD group shows a mean value of 1.64 mg/L and a median of 1.0 mg/L, with maximum values reaching up to 8.5 mg/L. Figure 4 and Figure 5 show the relationship between the 25(OH)D concentrations and clinical score in both groups.

In the NVD group, 25(OH)D concentrationsare widely distributed across all clinical scores, ranging from approximately 30 to over 200 ng/mL, with no evident downward trend as clinical score increases. Median and interquartile ranges remain within the optimal zone, and even patients with higher clinical scores (5–7) frequently exhibit serum 25(OH)D concentrations above 50 ng/mL.

In contrast, the LVD group shows consistently low serum 25(OH)D concentrations across all clinical scores, largely confined between 5 and 30 ng/mL, with a clear negative trend demonstrated by the regression line, indicating progressively lower vitamin D concentrations with increasing clinical score. Higher clinical scores are associated with a clustering of values in the moderate-to-severe deficiency range, including multiple observations below 10 ng/mL.

**Figure 6:**
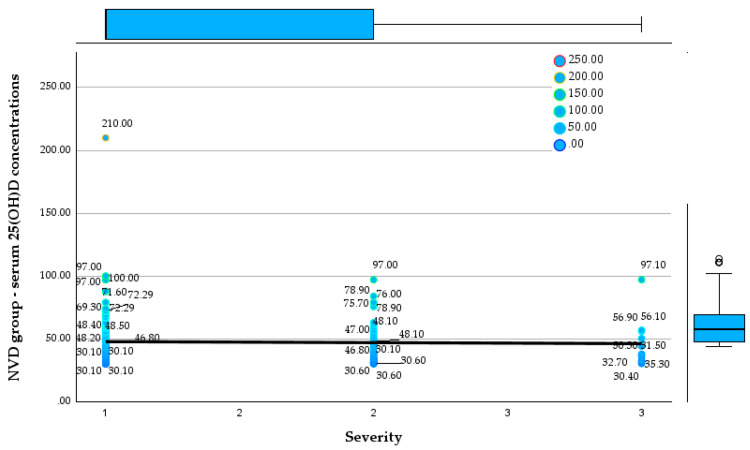
Relationship between serum 25(OH)D concentrations and disease severity in the NVD group.

**Figure 7:**
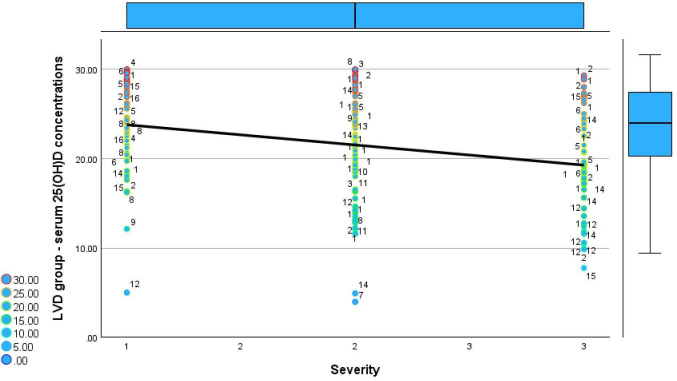
Relationship between serum 25(OH)D concentrations and disease severity in the LVD group.

Comparison of serum 25(OH)D concentrations across disease severity categories reveals fundamentally different patterns between the NVD and LVD groups. Figure 6 and Figure 7 show the relationship between serum 25(OH)D concentrations and disease severity in both groups.

In the NVD group, serum 25(OH)D concentrations remain consistently within the optimal range across mild, moderate, and severe disease, with median values clustering around 45–55 ng/mL and no meaningful decline with increasing severity. Even in severe cases, serum 25(OH)D concentrations frequently exceed 50 ng/mL, and extremely high values (up to 210 ng/mL) are observed, indicating preserved or augmented vitamin D status irrespective of clinical severity.

In contrast, the LVD group displays uniformly low serum 25(OH)D concentrations across all severity categories, with values largely confined between approximately 5 and 30 ng/mL. A clear inverse trend is evident, with progressively lower 25(OH)D concentrations from mild to severe disease, as reflected by the downward-sloping regression line and the increasing clustering of values in the moderate-to-severe deficiency range in severe cases. Notably, severe cases in the LVD group show the lowest concentrations, frequently below 15 ng/mL, whereas no patient in this group reaches the optimal range at any severity level.

### 3.2. Biological Markers and Hematologic Status

**Figure 8:**
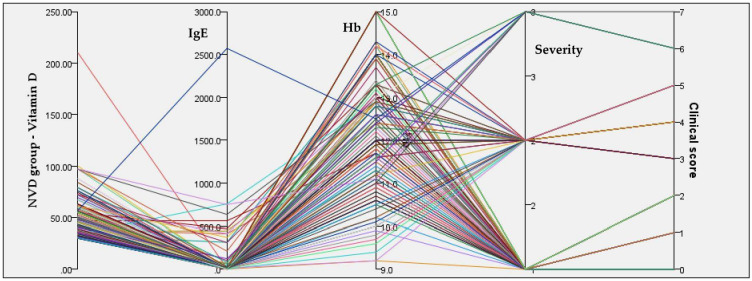
Integrated relationships between serum 25(OH)D concentrations, IgE, Hb, disease severity, and clinical score in the NVD group.

**Figure 9:**
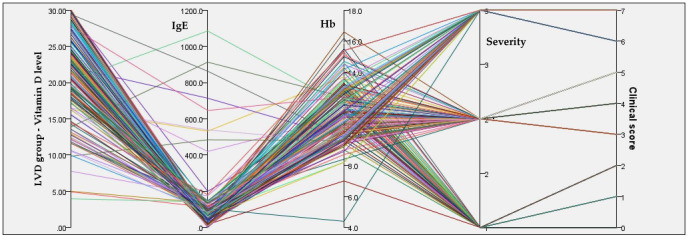
Integrated relationships between serum 25(OH)D concentrations, IgE, Hb, disease severity, and clinical score in the LVD group.

Total IgE levels in the NVD group showed a mean value of 52.85 IU/mL, but with an extremely wide range (0.4–2572 IU/mL), indicating a markedly heterogeneous population from an allergologic and immunologic perspective. Figure 8 and Figure 9 show the relationships between serum 25(OH)D concentrations, IgE, Hb, disease severity, and clinical score in both groups.

In the NVD group, concentrations span a wide range, predominantly within the sufficient to high range, and do not show a consistent alignment with higher disease severity or clinical scores. Lines corresponding to mild, moderate, and severe cases intersect broadly across Vitamin D, IgE, and Hb axes. Hemoglobin levels in the NVD group had a mean of 12.4 g/dL, with relatively limited dispersion (9.2–15.0 g/dL).

This group demonstrates a much more constrained and pathological pattern, with serum 25(OH)D concentrations tightly clustered at low values across nearly all patients.

### 3.3. Age-Stratified Comparison of Serum 25(OH)D Concentrations Between Groups

In early childhood (ages 1–3 years), marked and consistent differences in serum vitamin D status were observed between the NVD and LVD groups. In the NVD group, children aged ≤ 1 year (*n* = 82), 2 years (*n* = 20), and 3 years (*n* = 14) exhibited serum 25(OH)D concentrations, with mean values ranging from 44.0 to 50.8 ng/mL, and all measurements exceeded 30 ng/mL. No cases of vitamin D deficiency (<20 ng/mL) or severe deficiency were recorded in any of these age categories, and a substantial proportion of values exceeded 50 ng/mL, particularly in 1-year-old children (maximum 210.0 ng/mL). In contrast, the LVD group demonstrated a profoundly different profile from the earliest ages. Among children aged ≤ 1 year (*n* = 55), 2 years (*n* = 24), and 3 years (*n* = 9), all vitamin D values were below the optimal threshold of 50 ng/mL, with a large proportion falling below 25 ng/mL, consistent with severe deficiency. No child in the LVD group within these age categories reached optimal serum 25(OH)D concentrations, indicating an early and generalized deficit.

**Figure 10:**
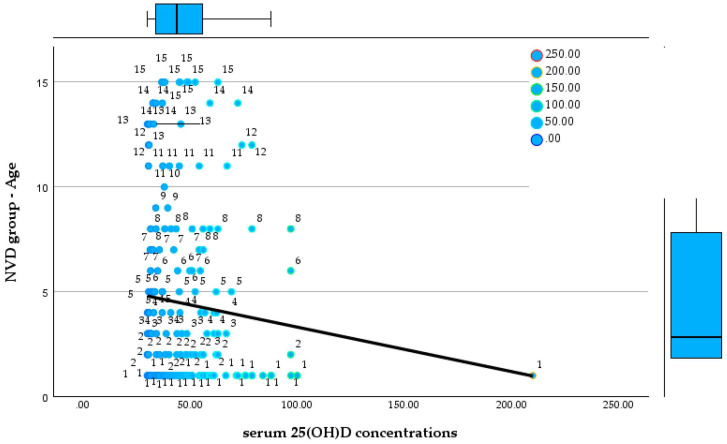
Relationship between serum 25(OH)D concentrations and age in the NVD group.

**Figure 11:**
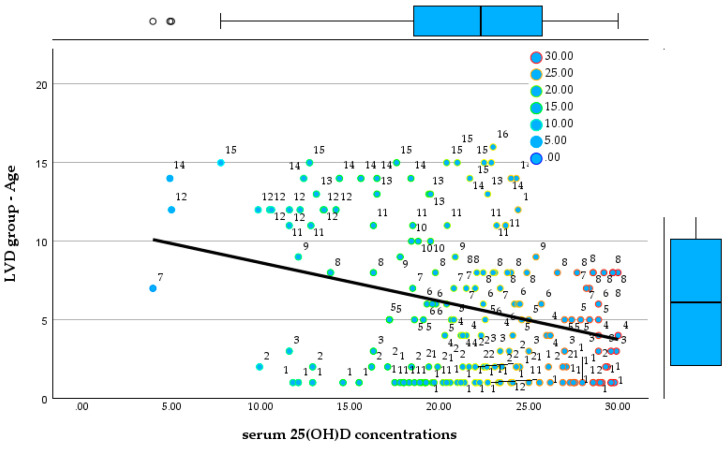
Relationship between serum 25(OH)D concentrations and age in the LVD group.

This divergence persisted throughout preschool ages (4–6 years). In the NVD group, children aged 4 years (*n* = 13), 5 years (*n* = 12), and 6 years (*n* = 10) continued to show stable and adequate vitamin D status, with mean values approximately between 45 and 46 ng/mL, and all individual measurements remaining above 30 ng/mL. No cases of moderate or severe deficiency were identified, and distributions remained relatively homogeneous, with several values exceeding 50 ng/mL. Conversely, children in the LVD group aged 4 years (*n* = 10), 5 years (*n* = 14), and 6 years (*n* = 10) exhibited persistently low serum 25(OH)D concentrations, ranging approximately from 17 to 30 ng/mL. Across all three age groups, vitamin D deficiency was universal, with multiple cases of severe deficiency (<25 ng/mL) and no values approaching the optimal range. Figure 10 and Figure 11 show the relationship between serum 25(OH)D concentrations and age in both groups.

Among school-aged children (7–9 years), the contrast between groups became even more pronounced. In the NVD group, children aged 7 years (*n* = 8), 8 years (*n* = 9), and 9 years (*n* = 6) maintained serum 25(OH)D concentrations exclusively within the optimal range, generally between 33 and 60 ng/mL, with no cases of deficiency identified. In contrast, the LVD group showed persistent and often severe vitamin D deficiency. At 7 years of age (*n* = 8), values ranged from as low as 3.96 ng/mL to 28.4 ng/mL, including an extremely severe deficiency. Similar patterns were observed at 8 years (*n* = 18; range 13.9–29.99 ng/mL) and 9 years (*n* = 4; range 12.1–25.4 ng/mL), where the majority of values fell within the severe deficiency range.

In late childhood and early adolescence (ages 10–15 years), the separation between the two cohorts remained absolute. In the NVD group, children aged 10 to 15 years consistently exhibited 25(OH)D concentrations above 30 ng/mL, with no recorded cases of deficiency or severe deficiency, despite smaller sample sizes in some age categories. In contrast, all LVD group participants within the same age range (ages 10–15 years; n ranging from 3 to 11 per group) demonstrated vitamin D deficiency, most frequently severe. Recorded values ranged from 4.91 ng/mL to 28.6 ng/mL, including extremely low concentrations at ages 10, 12, and 14 years. No LVD group patient in adolescence reached optimal serum 25(OH)D concentrations.

## 4. Discussion

Our study investigated the relationship between serum 25(OH)D concentrations and hospitalization duration among pediatric patients admitted to two tertiary medical institutions in Constanța County. The results revealed a statistically significant inverse correlation: children with lower concentrations had longer hospital stays. These findings support the hypothesis that hypovitaminosis D may contribute to prolonged recovery or increased disease severity in pediatric populations.

Beyond its immune-regulatory function, vitamin D exerts pleiotropic effects relevant to non-infectious pediatric conditions. It modulates skeletal muscle performance and cellular energy metabolism, promoting faster mobilization and rehabilitation during recovery. Vitamin D receptors are expressed in cardiac, neural, and endothelial tissues, influencing inflammatory cytokine cascades, oxidative stress, and tissue repair. Consequently, vitamin D deficiency may delay recovery in conditions such as anemia, malnutrition, or gastrointestinal disorders by impairing metabolic adaptation and epithelial healing. Furthermore, vitamin D contributes to circadian rhythm regulation and neuroendocrine stability, factors that may affect appetite, sleep, and stress responses, all of which can influence the course of hospitalization. Serum 25(OH)D concentrations were assessed only in patients with clinical suspicion of deficiency or related disorders, rather than through systematic screening of all admissions. This selective testing approach may introduce selection bias, potentially overrepresenting children with more severe clinical presentations. Nevertheless, this reflects real-world clinical practice and provides valuable insight into the subgroup of pediatric patients most likely to experience hypovitaminosis D–related complications.

The inverse association between serum 25(OH)D concentrations and hospitalization duration aligns with results reported by Bayramoğlu et al., who found that children with moderate-to-severe COVID-19 had significantly lower 25(OH)D levels and concurrently elevated inflammatory markers such as CRP and fibrinogen [18]. Their findings indicated a negative correlation between vitamin D status and systemic inflammation, as well as an increased likelihood of severe disease among vitamin D–deficient patients [18].

Total IgE concentrations in the NVD group had a mean value of 52.85 IU/mL but exhibited a markedly broad range (0.4–2572 IU/mL), reflecting substantial heterogeneity in the allergologic and immunologic profiles of the studied population. This pronounced variability is clinically relevant, as it indicates that the therapeutic response may differ according to the underlying immune status of individual patients. In the LVD group, further analyses confirmed total IgE as a parameter significantly associated with serum 25(OH)D levels.

Although our cohort included children hospitalized for a broad range of conditions beyond COVID-19, the similarity in vitamin D–related patterns across different pathologies supports the concept of vitamin D as a broader immunomodulatory and protective role in pediatric illness. This interpretation is further supported by Doğan et al., who reported/confirmed that serum 25(OH)D concentrations were significantly lower in COVID-19 positive children compared with healthy controls, suggesting a general immunological vulnerability associated with hypovitaminosis D [19].

Hemoglobin levels in the NVD group showed a mean value of 12.4 g/dL, with a relatively narrow range (9.2–15.0 g/dL), indicating the absence of clinically significant anemia in most patients. This hematologic stability is clinically relevant, as it minimizes the potential confounding effect of nutritional deficiencies or anemia-related chronic conditions on clinical outcomes. In the LVD group, hemoglobin was subsequently included in the explanatory models.

Moreover, Jaybhaye et al. emphasized the high prevalence of vitamin D deficiency among children with recurrent respiratory infections, reporting that none of their affected pediatric patients were vitamin D sufficient [20]. This observation aligns with our findings of widespread suboptimal vitamin D status across diverse clinical presentations. Their conclusion that vitamin D assessment should be incorporated into the management of children with recurrent illnesses is reinforced by our results, which demonstrate an association between deficiency and prolonged hospitalization irrespective of the specific diagnosis. While previous studies focused on specific infection entities such as SARS-CoV-2 or recurrent bronchitis, our cohort encompassed a wider range of diagnoses. diagnostic range. Despite this heterogeneity, a consistent association between lower concentrations and less favorable clinical outcomes has been reported across studies [21,22,23]. Our findings extend this evidence by demonstrating that vitamin D status remains relevant even in a multi-etiology hospital setting. Vitamin D appears to play a role in paediatric sickness resistance throughout these data. It may modulate inflammation, enhance innate immunity, or regulate tissue healing and systemic homeostasis. Regional literature increasingly emphasises vitamin D screening and supplementation as a simple, scalable intervention to minimize child hospitalisation time due to its low cost, accessibility, and safety.

## 5. Conclusions

In this retrospective cohort of pediatric patients hospitalized for respiratory tract infections, serum Vitamin D status was strongly associated with clinical/disease severity and hospitalization duration. Children with insufficient or deficient serum 25(OH)D concentrations experienced longer hospital stays compared with those with sufficient levels (mean 4.68 vs. 2.89 days) and presented with higher clinical severity scores (mean 3.77 vs. 1.62). LVD group also showed a greater inflammatory burden, reflected by higher CRP levels (mean 3.50 vs. 1.64 mg/L), increased frequency of fever (61.0% vs. 32.0%), and a substantially higher requirement for oxygen therapy (69.5% vs. 21.0%). Across all age categories, lower concentrations were inversely associated with hospitalization duration, disease severity, and clinical score.

## Data Availability

Data is available on request to the corresponding author.

## Supplementary Materials

The following supporting information can be downloaded at: https://www.mdpi.com/article/10.3390/reports9010054/s1.

## References

[1] Nagaria T.D., Shinde R.K., Shukla S., Acharya S., Acharya N., Jogdand S.D. (2023). The Sunlight-Vitamin D Connection: Implications for Patient Outcomes in the Surgical Intensive Care Unit. Cureus.

[2] Waldemer-Streyer R.J., Kim D., Chen J. (2022). Muscle cell-derived cytokines in skeletal muscle regeneration. FEBS J..

[3] Coman M., Hîncu M., Surlin P., Mateescu G., Nechita A., Banu M. (2011). Comparative histomorphometric study of bone tissue synthesized after electric and ultrasound stimulation. Rom. J. Morphol. Embryol..

[4] Raju A., Luthra G., Shahbaz M., Almatooq H., Foucambert P., Esbrand F.D., Zafar S., Panthangi V., Cyril Kurupp A.R., Khan S. (2022). Role of Vitamin D Deficiency in Increased Susceptibility to Respiratory Infections Among Children: A Systematic Review. Cureus.

[5] Pawlus Z., Mosiołek P., Bierć K., Pilśniak A., Janoska-Gawrońska A., Holecki M. (2025). Vitamin D Status in Patients at the Department of Internal, Autoimmune, and Metabolic Diseases—A Descriptive Cross-Sectional Study. Biomedicines.

[6] Radonsky V., Lazaretti-Castro M., Chiamolera M.I., Biscolla R.P.M., Lima J.V., Vieira J.G.H., Brandão C.M.A., Ramalho R.F., Maeda S.S., Cavichio M.W.E. (2024). Alert for the high prevalence of vitamin D deficiency in adolescents in a large Brazilian sample. J. Pediatr..

[7] (2017). Scientific Image and Illustration Software.

[8] Martens P.J., Gysemans C., Verstuyf A., Mathieu A.C. (2020). Vitamin D’s Effect on Immune Function. Nutrients.

[9] Giannini S., Giusti A., Minisola S., Napoli N., Passeri G., Rossini M., Sinigaglia L. (2022). The Immunologic Profile of Vitamin D and Its Role in Different Immune-Mediated Diseases: An Expert Opinion. Nutrients.

[10] Lungu C.N., Creteanu A., Mehedinti M.C. (2024). Endovascular Drug Delivery. Life.

[11] Ghiga G., Țarcă E., Țarcă V., Spoială E.L., Păduraru G., Gimiga N., Boca L.O., Iftinchi O., Donos M.A., Manole L.M. (2024). Vitamin D Deficiency: Insights and Perspectives from a Five-Year Retrospective Analysis of Children from Northeastern Romania. Nutrients.

[12] Badiu Tișa I., Cozma-Petruț A., Samașca G., Miere D., Filip L., Banc R., Mîrza O., Iancu M. (2024). Vitamin D Status among 2–18-Year-Old Romanian Pediatric Patients: A Single-Center Study. Nutrients.

[13] Margan M.M., Alexandru A., Ivan C.S., Boeriu E., Tanasescu S., Cârstea A.M., Varga N.I., Margan R., Cindrea A.C., Negrean R.A. (2025). Vitamin D Status in Children: Romania’s National Vitamin D Screening Programme in Context of the COVID-19 Pandemic. Med. Sci..

[14] Holick M.F., Binkley N.C., Bischoff-Ferrari H.A., Gordon C.M., Hanley D.A., Heaney R.P., Murad M.H., Weaver C.M., Endocrine Society (2011). Evaluation, treatment, and prevention of vitamin D deficiency: An Endocrine Society clinical practice guideline. J. Clin. Endocrinol. Metab..

[15] Chicco D., Sichenze A., Jurman G. (2025). A simple guide to the use of Student’s *t*-test, Mann-Whitney U test, Chi-squared test, and Kruskal-Wallis test in biostatistics. BioData Min..

[16] Singhal R., Rana R. (2015). Chi-square test and its application in hypothesis testing. J. Pract. Cardiovasc. Sci..

[17] Rovetta A. (2020). Raiders of the Lost Correlation: A Guide on Using Pearson and Spearman Coefficients to Detect Hidden Correlations in Medical Sciences. Cureus.

[18] Bayramoğlu E., Akkoç G., Ağbaş A., Akgün Ö., Yurdakul K., Selçuk Duru H.N., Elevli M. (2021). The association between vitamin D levels and the clinical severity and inflammation markers in pediatric COVID-19 patients: Single-center experience from a pandemic hospital. Eur. J. Pediatr..

[19] Doğan A., Dumanoğlu Doğan İ., Uyanık M., Köle M.T., Pişmişoğlu K. (2022). The Clinical Significance of Vitamin D and Zinc Levels with Respect to Immune Response in COVID-19 Positive Children. J. Trop. Pediatr..

[20] Jaybhaye A.P., Sangle A.L., Ugra D., Chittal R.Y. (2022). A Hospital-Based Study of Vitamin D Levels in Children with Recurrent Respiratory Infections. Cureus.

[21] Liu Y., Clare S., D’Erasmo G., Heilbronner A., Dash A., Krez A., Zaworski C., Haseltine K., Serota A., Miller A. (2023). Vitamin, D and SARS-CoV-2 Infection: SERVE Study (SARS-CoV-2 Exposure and the Role of Vitamin D among Hospital Employees). J. Nutr..

[22] Najih M., Boussettine R., El Kehel M.S., Nabil K., Azmi H., Berradi H., Ennaji M.M. (2025). Impact of Vitamin D Levels on Clinical Outcomes in SARS-CoV-2 Infections. Cureus.

[23] Sartini M., Del Puente F., Carbone A., Schinca E., Ottria G., Dupont C., Piccinini C., Oliva M., Cristina M.L. (2024). The Effect of Vitamin D Supplementation Post COVID-19 Infection and Related Outcomes: A Systematic Review and Meta-Analysis. Nutrients.

