# Supplementary material for: Associations Between Serum 25(OH)D Concentrations and Clinical Characteristics in Pediatric Patients
Source: Reports (MDPI). 2026 Feb 9;9(1):54. doi: 10.3390/reports9010054 (PMC12921969; doi:10.3390/reports9010054)
Supplement: Supplementary file 1 [file reports-09-00054-s001.zip › reports-4103178-supplementary.pdf]

1. Population characteristics

CONTROL group

Sex

	N	%
male	103	51.5%
female	97	48.5%

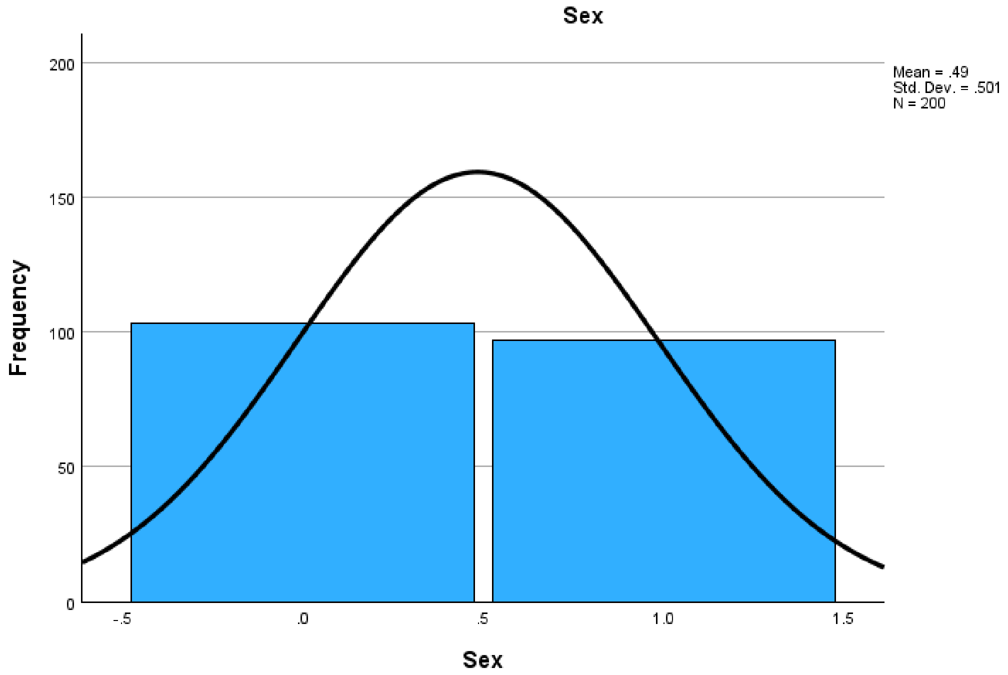

Environment

	N	%
urban	133	66.5%
rural	67	33.5%

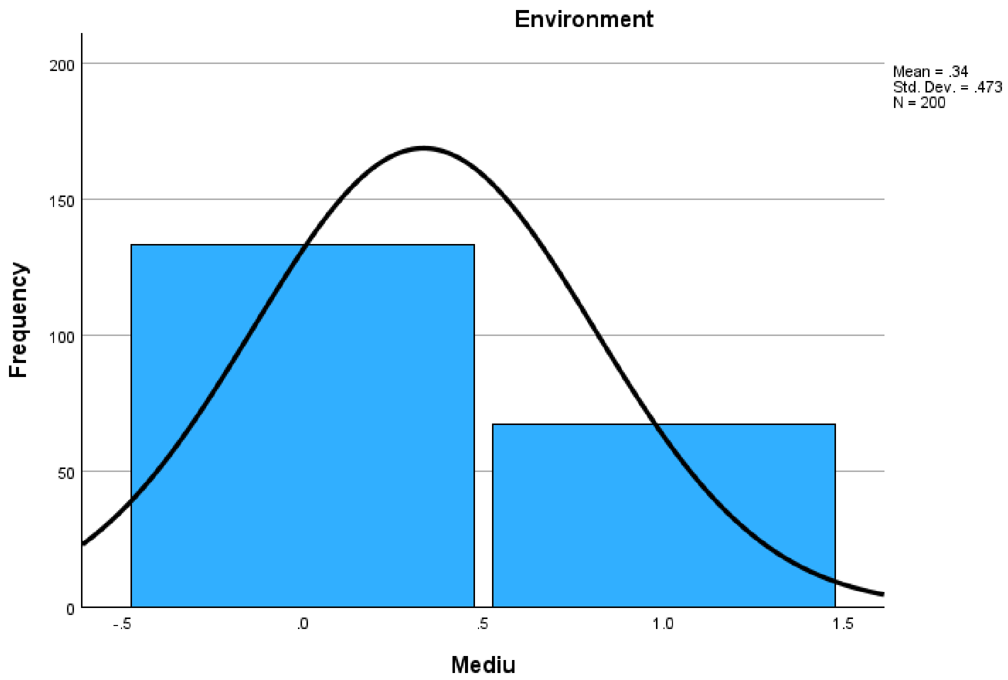

Ethnic_background

	N	%
Romanian	142	71.0%
Tatar	6	3.0%
Turkish	15	7.5%
Macedonean	35	17.5%
Ukrainean	2	1.0%

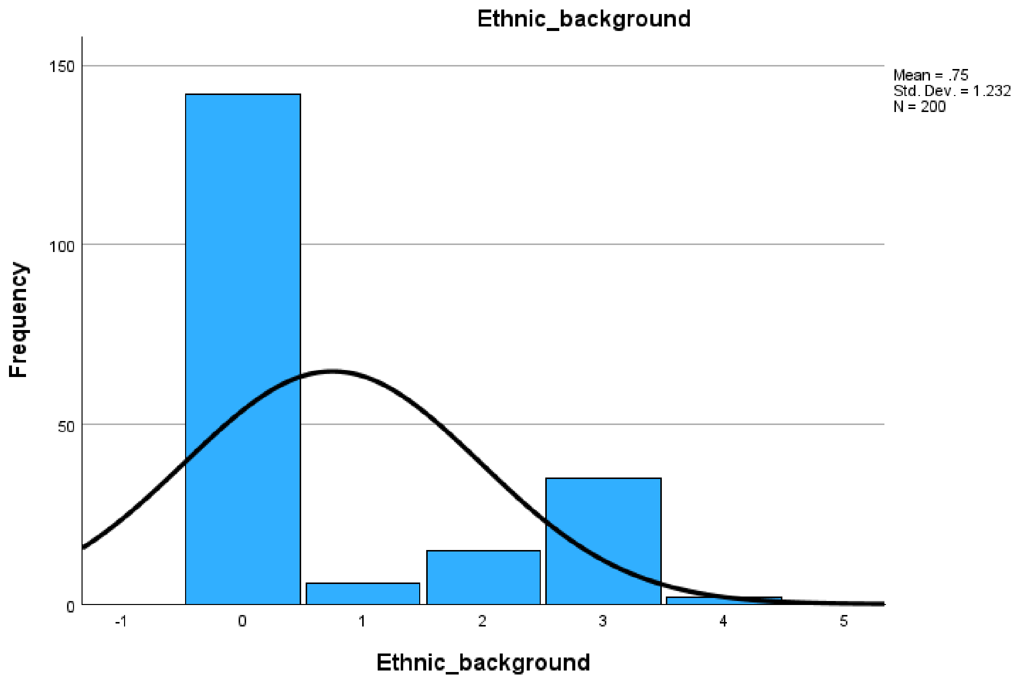

Season

	N	%
autumn	72	36.0%
winter	18	9.0%
spring	33	16.5%
summer	77	38.5%

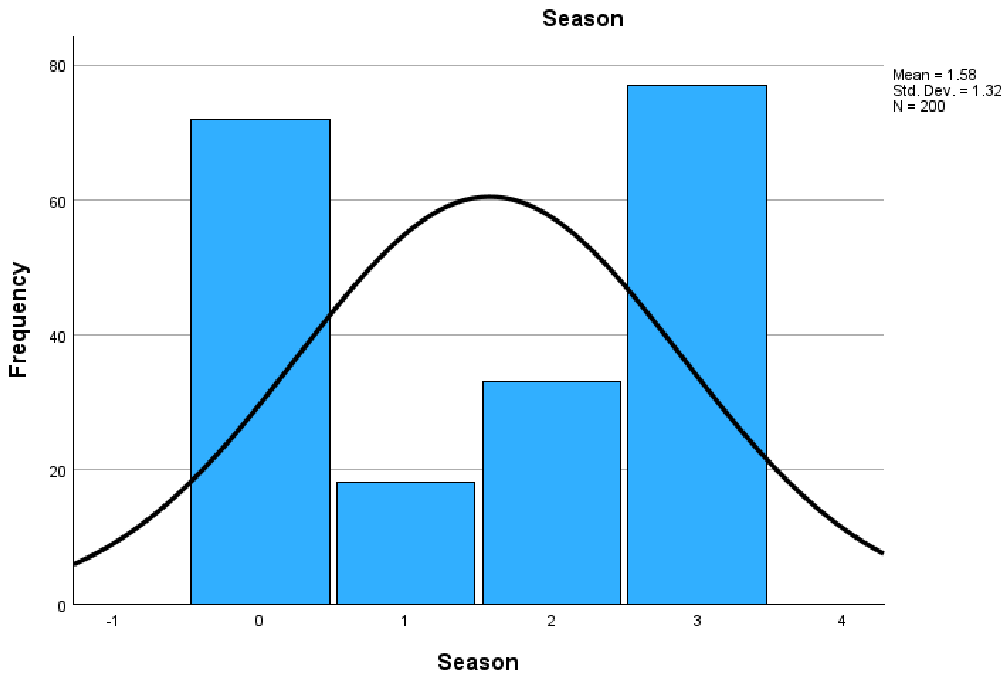

Vitamin_D_supplementation

	N	%
no	98	49.0%
yes	102	51.0%

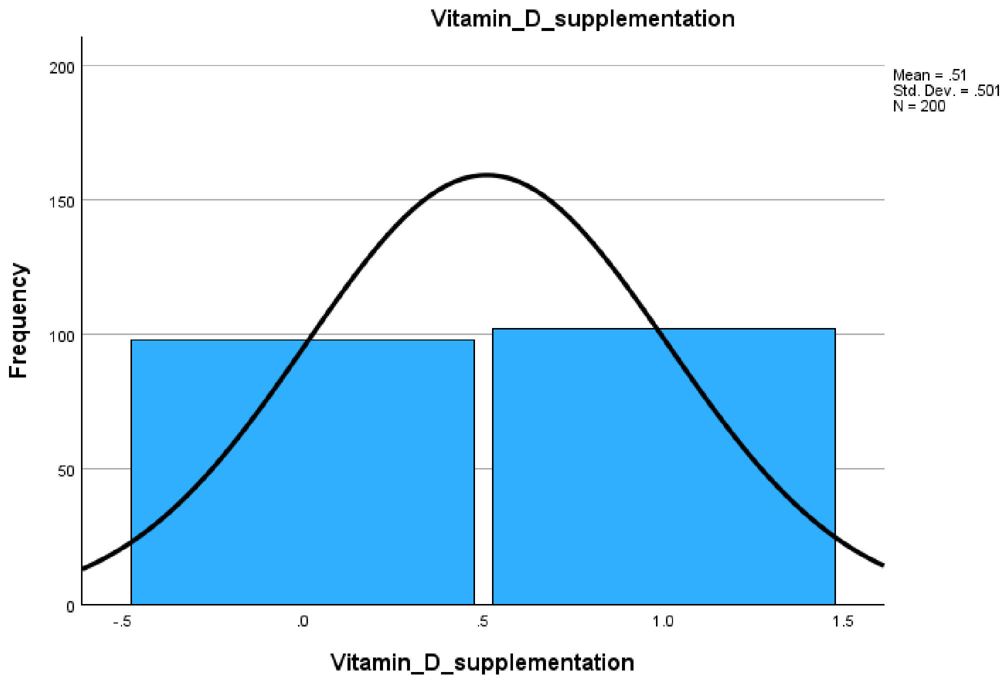

O2_therapy

	N	%
no	158	79.0%
yes	42	21.0%

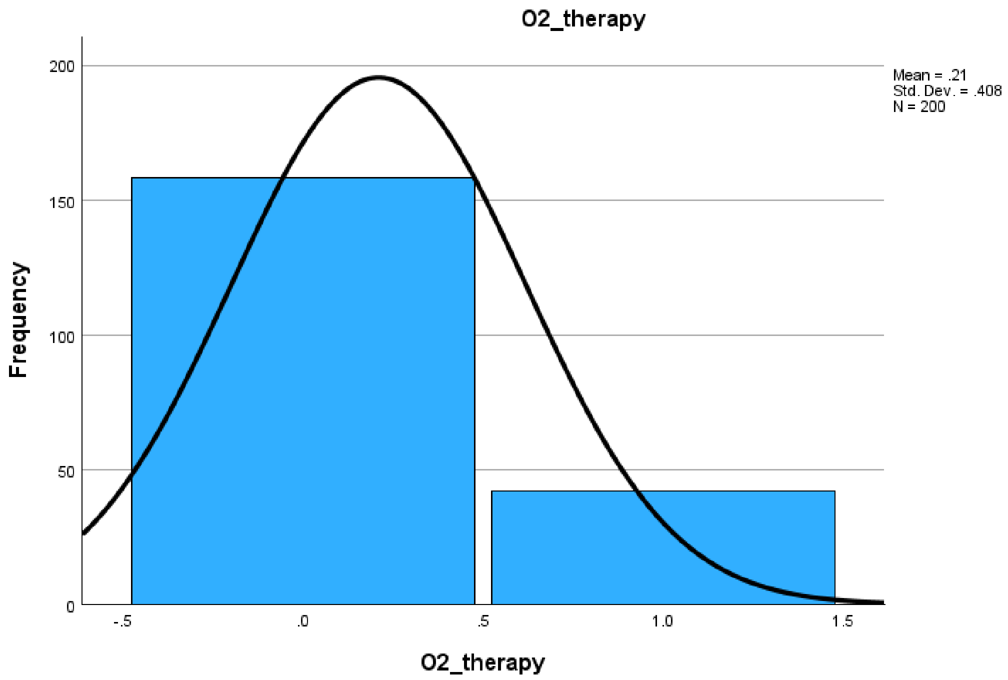

Fever

	N	%
no	136	68.0%
yes	64	32.0%

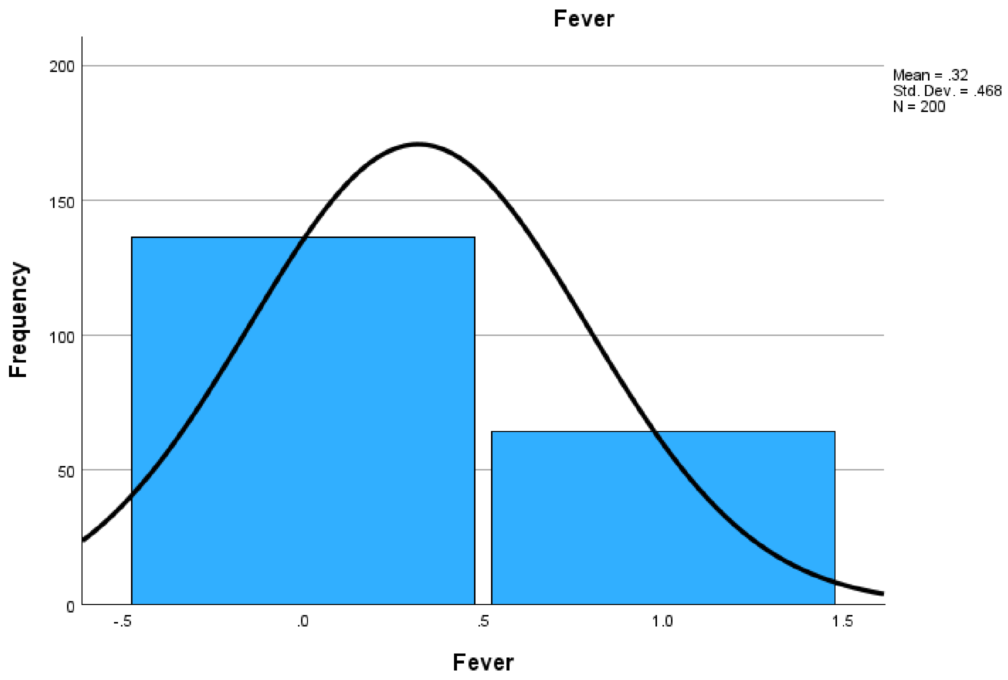

Severity

	N	%
mild	145	72.5%
moderate	44	22.0%
severe	11	5.5%

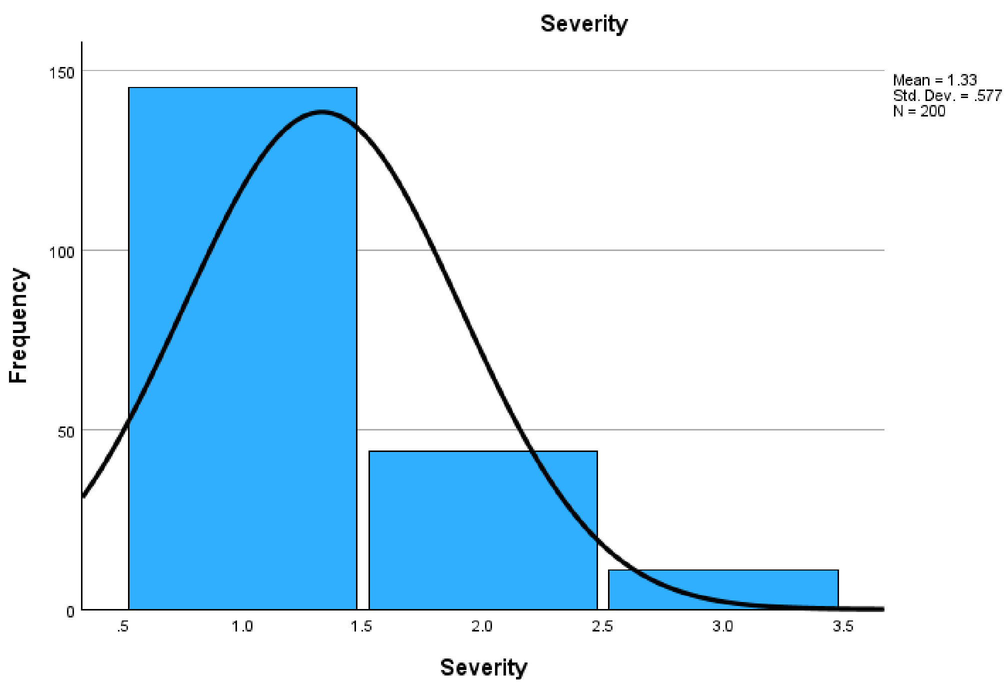

INTERVENTION group

Sex

	N	%
male	118	59.0%
female	82	41.0%

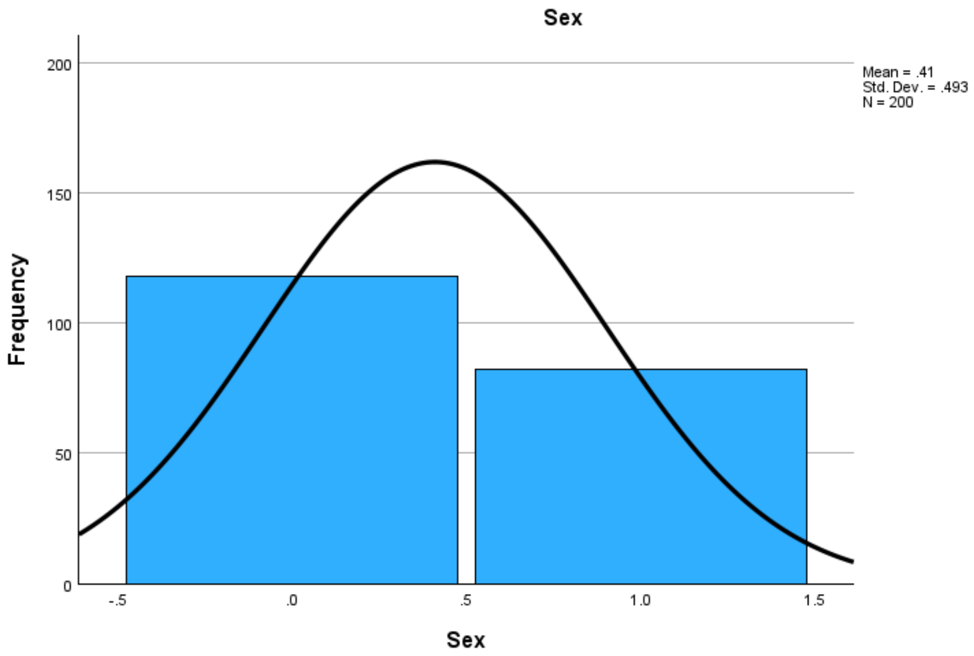

Environment

	N	%
urban	135	67.5%
rural	65	32.5%

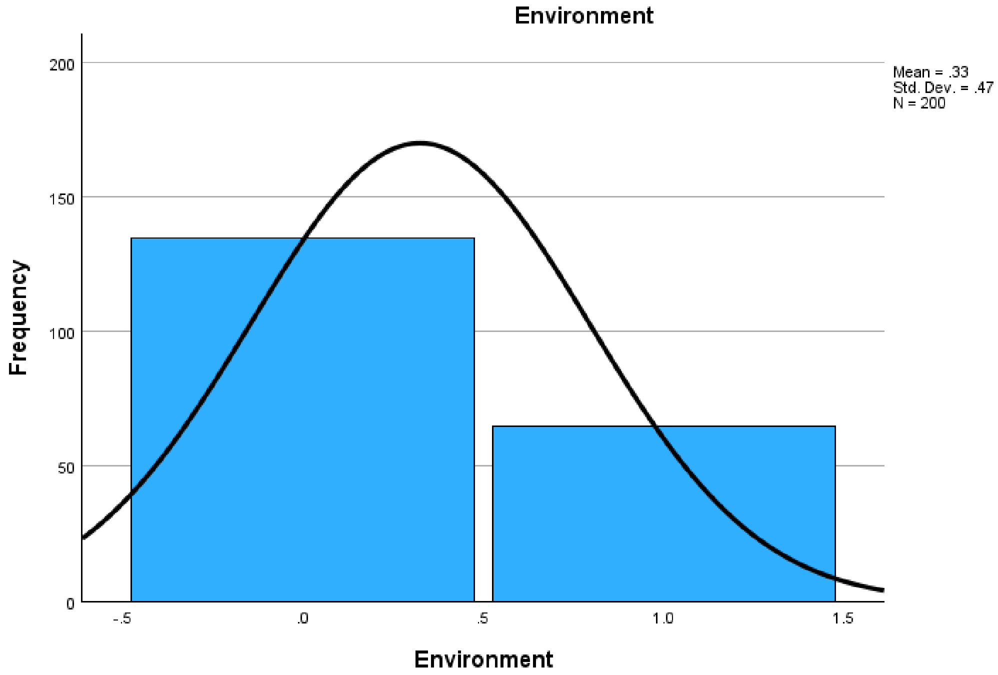

Ethnic_background

	N	%
Romanian	141	70.5%
Tatar	16	8.0%
Turkish	18	9.0%
Macedonian	23	11.5%
Ukrainean	2	1.0%

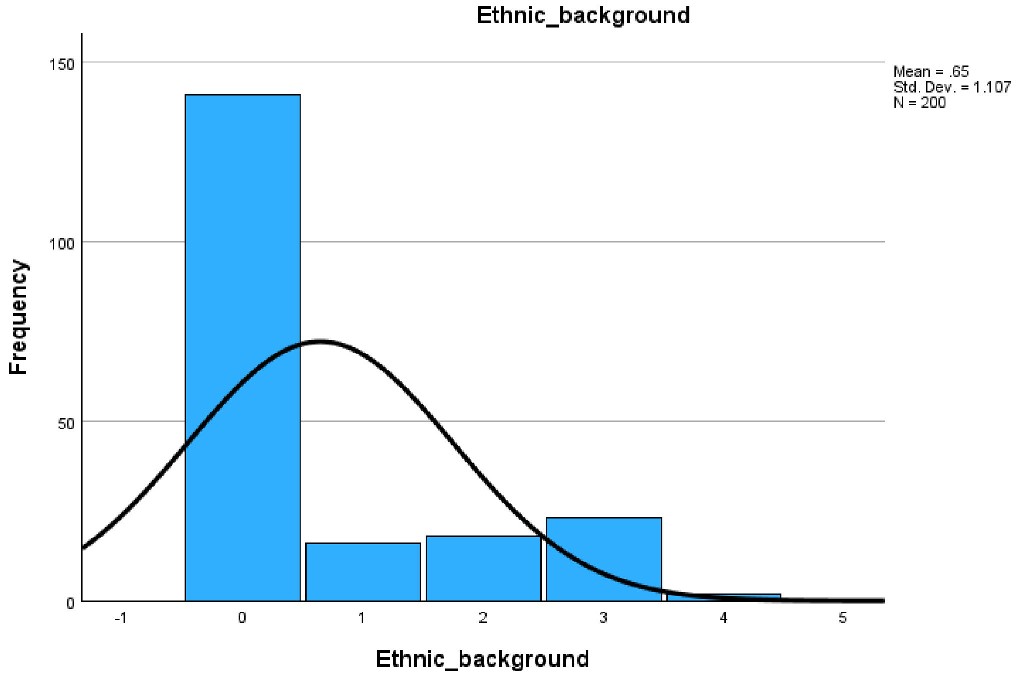

Season

	N	%

autumn	52	26.0%
winter	47	23.5%
spring	55	27.5%
summer	46	23.0%

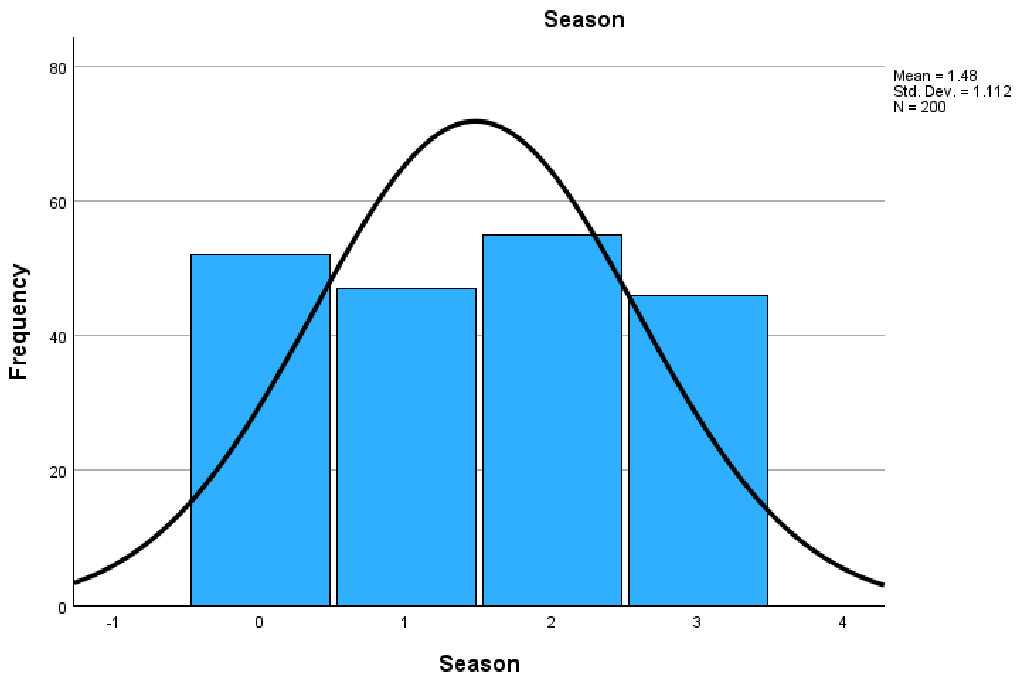

Vitamin_D_supplementation

	N	%
no	133	66.5%
yes	67	33.5%

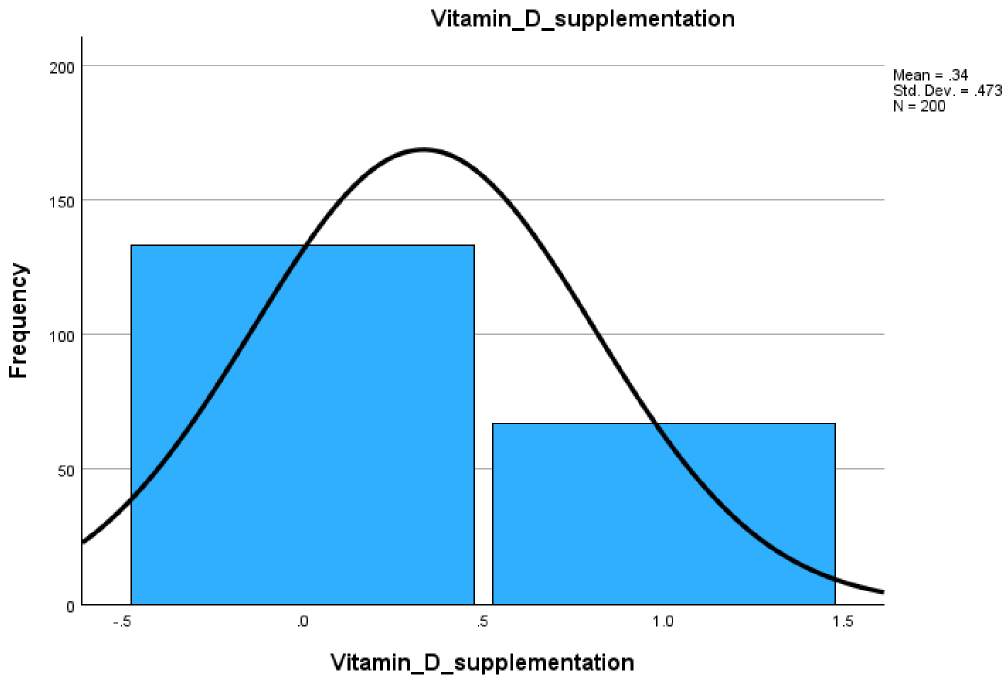

O2_therapy

	N	%
no	78	39.0%
yes	122	61.0%

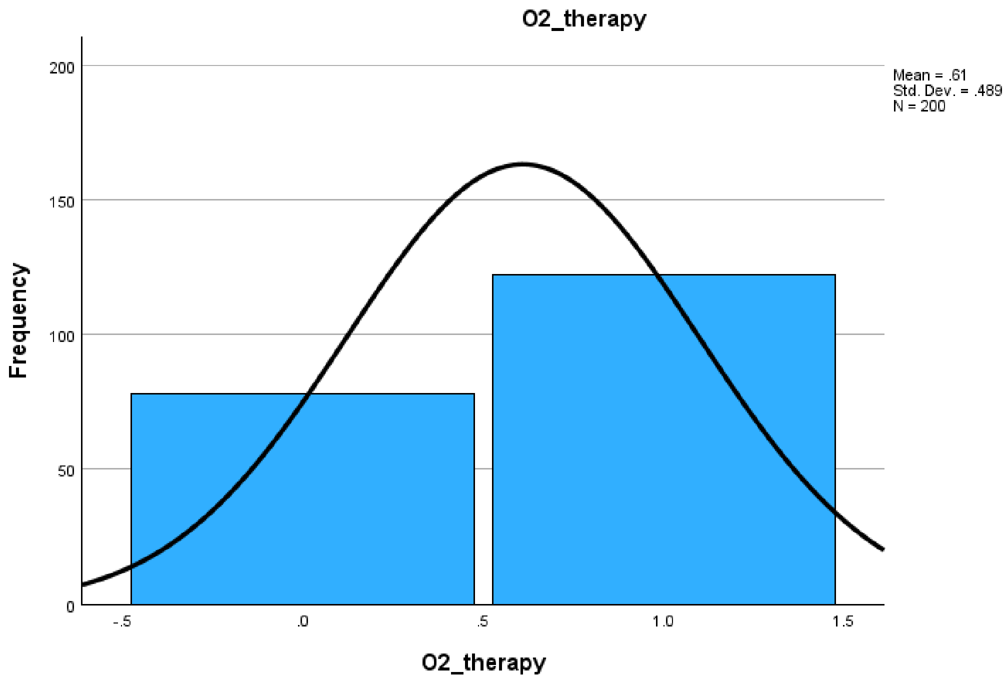

Fever

	N	%
no	61	30.5%
yes	139	69.5%

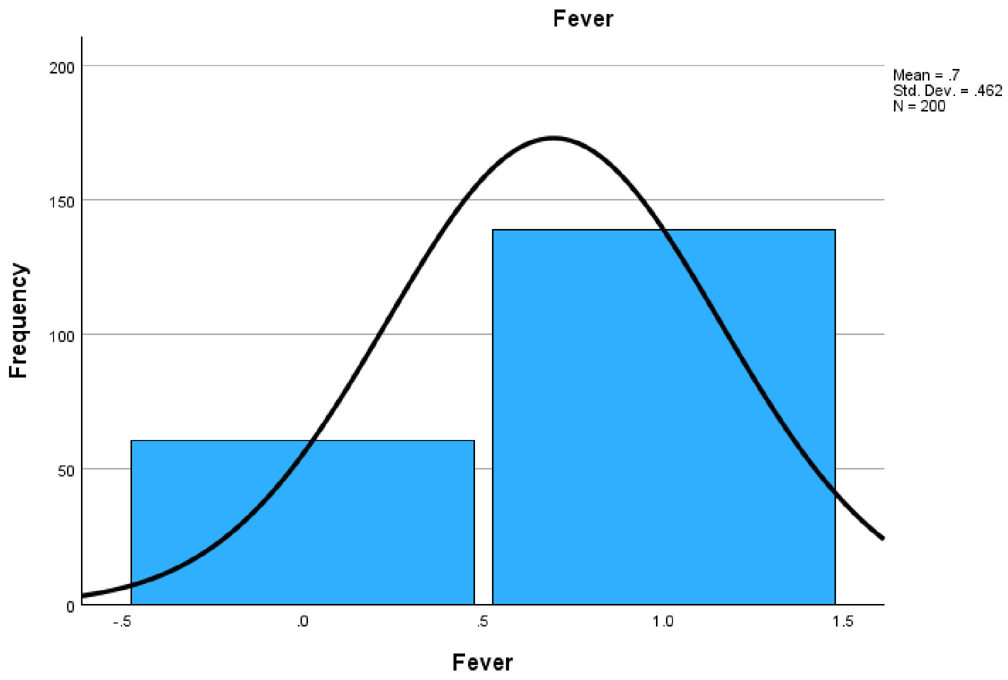

Severity

	N	%
mild	63	31.5%
moderate	84	42.0%
severe	53	26.5%

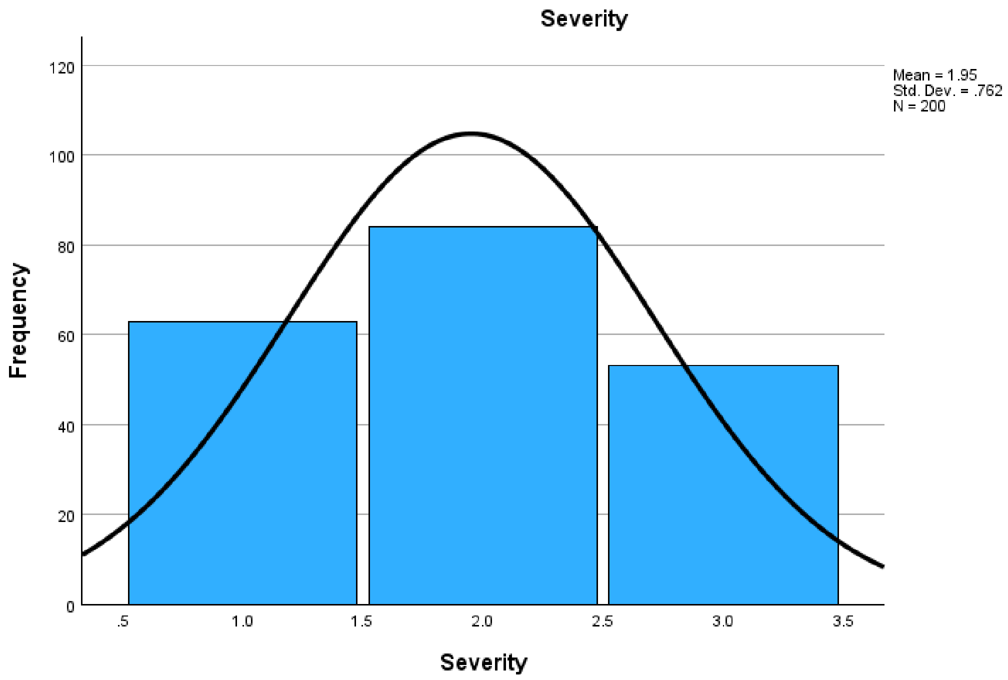

Case Processing Summary CONTROL group

		Severity	Clinical score	CRP	IgE	O2 therapy	Days of hospitalisation
Series or Sequence Length		200	200	200	200	200	200
Number of Missing Values in the Plot	Negative or Zero Before Log Transform	0	78[a]	0	0	158[a]	0
	User-Missing	0	0	0	0	0	0
	System-Missing	0	0	0	0	0	0

a. The minimum value is .000.

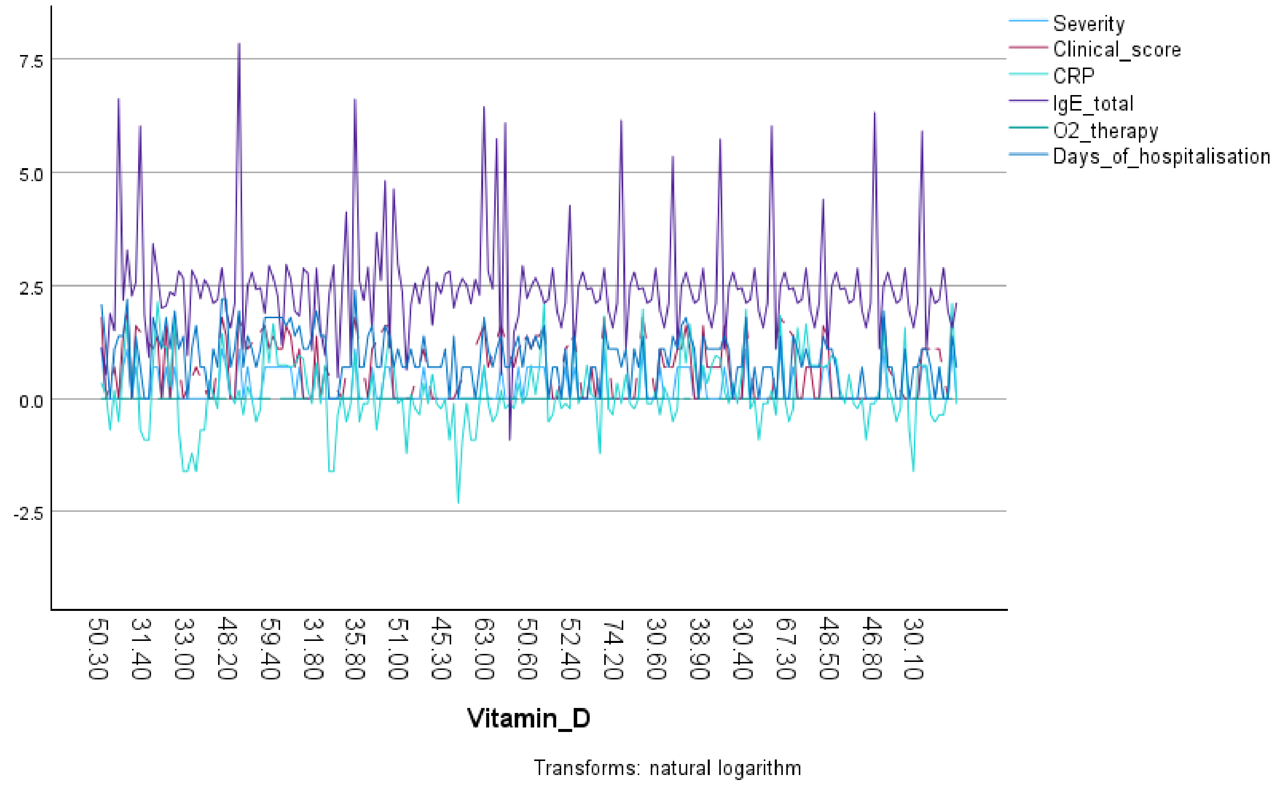

Case Processing Summary CONTROL group

		Age	Sex	Environment	Ethnic background	Season	Vitamin D supplementation
Series or Sequence Length		200	200	200	200	200	200
Number of Missing Values in the Plot	Negative or Zero Before Log Transform	0	103[a]	133[a]	142[a]	72[a]	98[a]
	User-Missing	0	0	0	0	0	0

	System-Missing	0	0	0	0	0	0

a. The minimum value is .000.

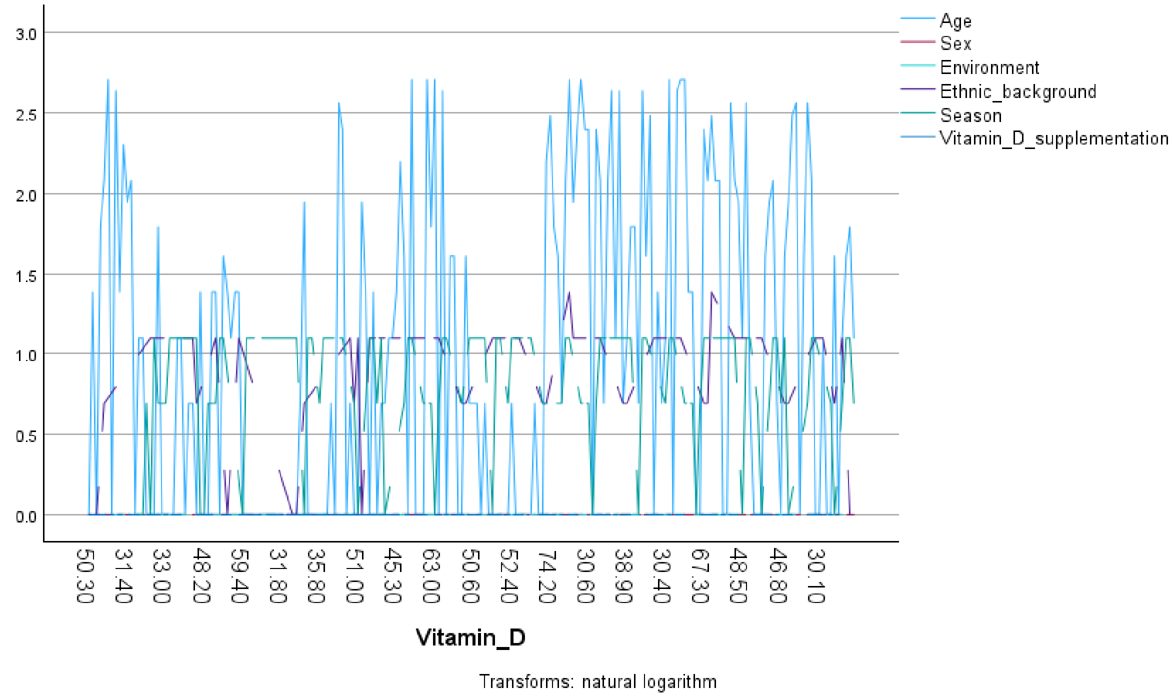

Case Processing Summary INTERVENTION group

		Days of hospitalisation	CRP	O2 therapy	Clinical score	Severity	IgE
Series or Sequence Length		200	200	200	200	200	200
Number of Missing Values in the Plot	Negative or Zero Before Log Transform	0	0	78[a]	28[a]	0	0
	User-Missing	0	0	0	0	0	0
	System-Missing	0	0	0	0	0	0

a. The minimum value is .000.

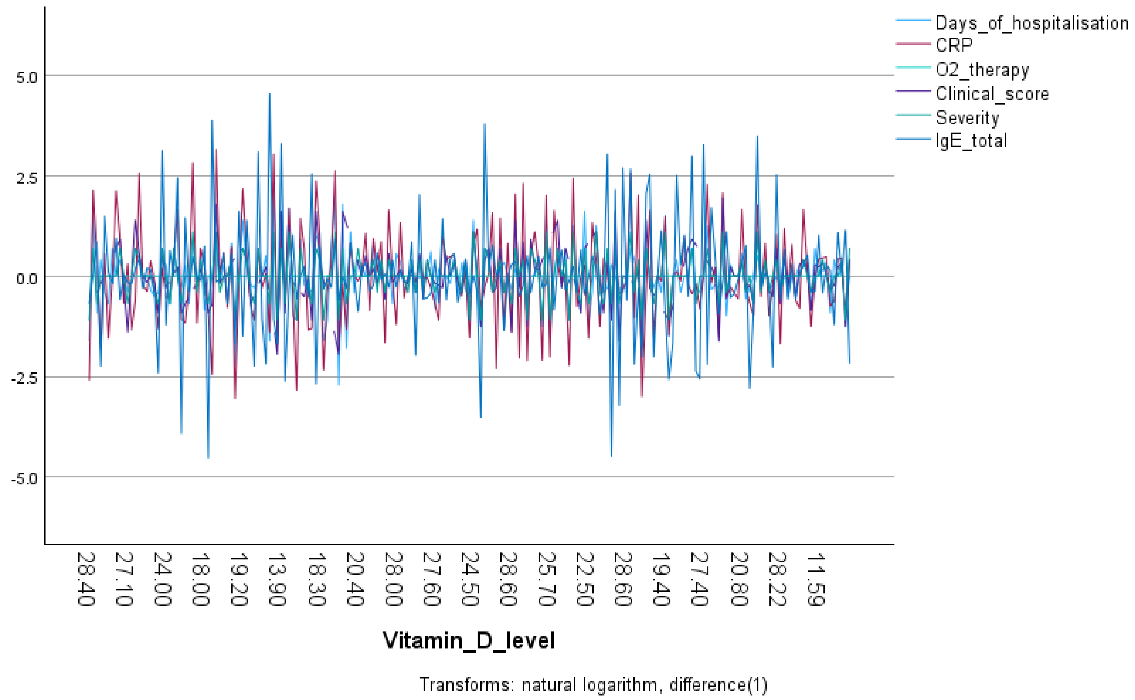

Case Processing Summary INTERVENTION group

		Age	Sex	Environment	Ethnic background	Season	Vitamin D supplementation
Series or Sequence Length		200	200	200	200	200	200
Number of Missing Values in the Plot	Negative or Zero Before Log Transform	0	118[a]	135[a]	141[a]	52[a]	133[a]
	User-Missing	0	0	0	0	0	0
	System-Missing	0	0	0	0	0	0

a. The minimum value is .000.

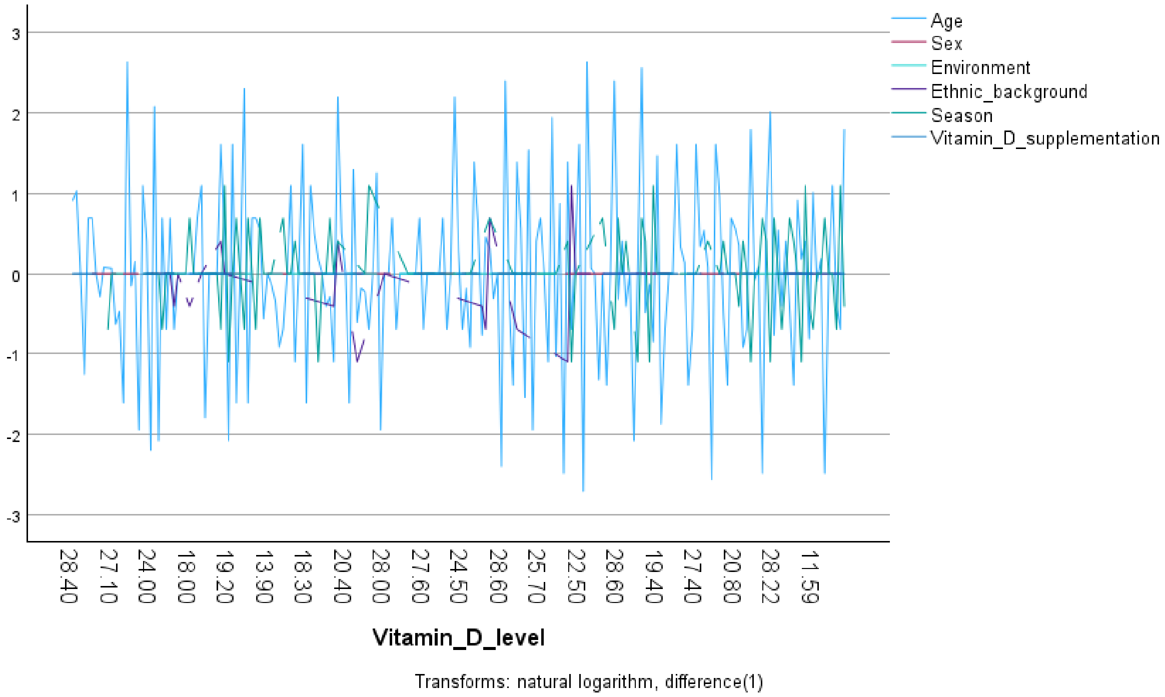
3
2
1
0
-1
-2
-3
Age
Sex
Environment
Ethnic_background
Season
Vitamin_D_supplementation
28.40
27.10
24.00
18.00
19.20
13.90
18.30
20.40
28.00
27.60
24.50
28.60
25.70
22.50
28.60
19.40
27.40
20.80
28.22
11.59
Vitamin_D_level
Transforms: natural logarithm, difference(1)
